# Hydroxychloroquine as an add-on therapy for the induction therapy of MPO-AAV: a retrospective observational cohort study

**DOI:** 10.1093/ckj/sfae264

**Published:** 2024-09-06

**Authors:** Yizi Gong, Ting Meng, Wei Lin, Xueling Hu, Rong Tang, Qi Xiong, Joshua D Ooi, Peter J Eggenhuizen, Jinbiao Chen, Ya-Ou Zhou, Hui Luo, Jia Xu, Ning Liu, Ping Xiao, Xiangcheng Xiao, Yong Zhong

**Affiliations:** Department of Nephrology, Xiangya Hospital, Central South University, Changsha, Hunan Province, China; Key Laboratory of Biological Nanotechnology of National Health Commission, Xiangya Hospital, Central South University, Changsha, Hunan, China; National Clinical Research Center for Geriatric Disorders, Xiangya Hospital, Central South University, Changsha, Hunan, China; Hunan International Scientific and Technological Cooperation Base of Diagnosis and Treatment for ANCA Associated Vasculitis, Changsha, Hunan Province, China; Department of Nephrology, Xiangya Hospital, Central South University, Changsha, Hunan Province, China; Department of Pathology, Xiangya Hospital, Central South University, Changsha, Hunan Province, China; Department of Nephrology, Xiangya Hospital, Central South University, Changsha, Hunan Province, China; Department of Nephrology, Xiangya Hospital, Central South University, Changsha, Hunan Province, China; Department of Nephrology, Xiangya Hospital, Central South University, Changsha, Hunan Province, China; Department of Nephrology, Xiangya Hospital, Central South University, Changsha, Hunan Province, China; Centre for Inflammatory Diseases, Department of Medicine, School of Clinical Sciences, Monash University, Clayton, VIC, Australia; Centre for Inflammatory Diseases, Department of Medicine, School of Clinical Sciences, Monash University, Clayton, VIC, Australia; Department of medical records & information, Xiangya Hospital, Central South University, Changsha, Hunan Province, China; Department of Rheumatology and Immunology, Xiangya Hospital, Central South University, Changsha, China; Department of Rheumatology and Immunology, Xiangya Hospital, Central South University, Changsha, China; Department of Nephrology, Xiangya Hospital, Central South University, Changsha, Hunan Province, China; Department of Nephrology, Xiangya Hospital, Central South University, Changsha, Hunan Province, China; Department of Nephrology, Xiangya Hospital, Central South University, Changsha, Hunan Province, China; Department of Nephrology, Xiangya Hospital, Central South University, Changsha, Hunan Province, China; Key Laboratory of Biological Nanotechnology of National Health Commission, Xiangya Hospital, Central South University, Changsha, Hunan, China; National Clinical Research Center for Geriatric Disorders, Xiangya Hospital, Central South University, Changsha, Hunan, China; Hunan International Scientific and Technological Cooperation Base of Diagnosis and Treatment for ANCA Associated Vasculitis, Changsha, Hunan Province, China; Department of Nephrology, Xiangya Hospital, Central South University, Changsha, Hunan Province, China; Key Laboratory of Biological Nanotechnology of National Health Commission, Xiangya Hospital, Central South University, Changsha, Hunan, China; National Clinical Research Center for Geriatric Disorders, Xiangya Hospital, Central South University, Changsha, Hunan, China; Hunan International Scientific and Technological Cooperation Base of Diagnosis and Treatment for ANCA Associated Vasculitis, Changsha, Hunan Province, China

**Keywords:** ANCA-associated vasculitis, hydroxychloroquine, myeloperoxidase-AAV, remission

## Abstract

**Background:**

The remission rate of myeloperoxidase (MPO)-antineutrophil cytoplasmic antibody (ANCA)-associated vasculitis (AAV) patients who received standard induction therapy is far from satisfactory. Improving the remission rate of MPO-AAV patients is essential. Hydroxychloroquine (HCQ), one of the classic antimalarial drugs, has been widely used in various autoimmune rheumatic diseases. This retrospective observational cohort study is aimed to evaluate the efficacy and safety of HCQ during induction treatment for MPO-AAV.

**Methods:**

The medical records of patients diagnosed with MPO-AAV at Xiangya Hospital, Central South University from January 2021 to September 2023 were collected. They were assigned to the HCQ group or control group according to whether they used HCQ. The patients included were screened by propensity score matching. To evaluate whether MPO-AAV patients benefited from HCQ, we compared the prognosis of the two groups. The adverse effects of HCQ during follow-up were recorded.

**Results:**

The composition ratio of complete remission, response and treatment resistance between HCQ group and control group were different statistically (*P *= .021). There was no significant difference between the two groups in 1-year renal survival (*P *= .789). The HCQ group had better 1-year patient survival than the control group (*P *= .049). No serious adverse effects were documented in the HCQ group.

**Conclusions:**

HCQ together with standard induction treatment may improve the remission rate of MPO-AAV patients, and HCQ had good safety in our study.

KEY LEARNING POINTS **What was known:**

- The remission rate of anti-neutrophil cytoplasmic antibody (ANCA)-associated vasculitis (AAV) after standard treatment was far from satisfactory.
- AAV patients who did not reach remission had a bad prognosis.
- To find a method to improve the remission rate of AAV, we conducted this study.

**This study adds:**

- Hydroxychloroquine (HCQ) together with standard induction treatment may improve the remission rate of MPO-AAV patients, and HCQ had good safety in our study.

**Potential impact:**

- MPO-AAV patients during induction therapy may be administered HCQ to improve disease conditions.

## INTRODUCTION

Anti-neutrophil cytoplasmic antibody (ANCA)-associated vasculitis (AAV) is a group of autoimmune disorders that could cause damage to multiple systems. Myeloperoxidase (MPO)-AAV and proteinase 3 (PR3)-AAV are the two main types of AAV according to the hallmark of AAV [1]. The prevalence of MPO-AAV is higher in China [2]. According to the latest recommendations of Kidney Disease: Improving Global Outcomes (KDIGO) and European League Against Rheumatism/European Renal Association–European Dialysis and Transplant Association (EULAR/ERA-EDTA) [3, 4], induction treatment comprises glucocorticoid and cyclophosphamide or rituximab. The remission rate of AAV patients was around 70%–90% with standard induction therapy [5–7]. The remission rate of MPO-AAV was even lower than that of PR3-AAV, and we recently reported that the remission rate of 184 MPO-AAV patients was 65.2% [8]. Moreover, the renal survival rate and patient survival rate of MPO-AAV patients who did not achieve remission after induction treatment were significantly worse. Thus, modified regimes are needed to improve the remission rate of AAV patients.

Used initially as an anti-malaria drug, hydroxychloroquine (HCQ) emerged in 1950 after the progress of chemical synthesis and structural modification of quinine, which was extracted from cinchona [9]. In 1975, for the first time, a study described improved cutaneous symptoms, reduced dosage of glucocorticoid and reduced relapse rate in patients with systemic lupus erythematosus (SLE) after taking HCQ [10]. Subsequent studies confirmed that HCQ would function well in modifying other rheumatic diseases [11]. In addition, HCQ was reported to have certain effects on reducing proteinuria in immunoglobulin A (IgA) nephropathy, relieving cutaneous disease and vasculitis, delaying the development of organ involvement in autoimmune diseases, and protecting against thrombosis, tumor or infection [11–14]. The major adverse event of HCQ was ocular lesions, such as retinal injuries [15]. In addition, the side effects of HCQ were slight, and most of these symptoms related to side effects can usually be relieved or disappear after discontinuing the use of HCQ [16, 17].

In summary, as an immune regulator with good safety, HCQ has additional antithrombotic, cardioprotective, antimicrobial and anti-neoplastic benefits which would be immensely valuable in patients with AAV who are at risk of infections, malignancy and thrombosis, owing to the disease itself and background immunosuppression. However, HCQ has not been widely used in AAV. Therefore, we conducted this retrospective observational cohort study to explore the efficacy and safety of the addition of HCQ to standard induction therapy in MPO-AAV.

## MATERIALS AND METHODS

### Study population

The cases in this study were derived from patients diagnosed with AAV at Xiangya Hospital, Central South University, China, from January 2021 to September 2023 [18]. We applied standard indirect immunofluorescence assay (IFA) (Euroimmun, Lübeck, Germany) and direct enzyme-linked immunosorbent assay (ELISA) (Inova Diagnostics, San Diego, CA, USA) to detect the presence of ANCA. The inclusion criteria were: (i) patients with new diagnosis of microscopic polyangiitis or granulomatosis with polyangiitis or renal limited vasculitis according to the 2012 Chapel Hill Consensus Conference and positive for MPO-ANCA; (ii) patients who were 18–85 years old; and (iii) patients with active disease (defined by at least one major or three minor Birmingham Vasculitis Activity Score (BVAS) 2003 items. The exclusion criteria were: (i) patients positive for PR3-ANCA or anti-glomerular basement membrane antibodies; (ii) the coexistence of other autoimmune diseases or nephropathy; (iii) previous malignancy; (iv) female patients who were at risk of pregnancy, pregnant or breastfeeding; (v) activated hepatitis B virus, hepatitis C virus or human immunodeficiency virus infection; and (vi) previous treatments with cyclophosphamide over 2 weeks.

The studies involving human participants were reviewed and approved by the Medical Ethics Committee of the Xiangya Hospital of Central South University (approval number 202207431). The patients/participants provided their written informed consent to participate in this study.

### Data retrieval

The baseline clinical characteristics, laboratory test data, and therapeutic regimen of eligible patients were obtained from the data in electronic medical records. BVAS (version 3) was used to estimate disease activity [19]. All patients received glucocorticoids combined with immunosuppressive drugs for induction treatment. Patients were assigned to HCQ group or control group according to whether they took HCQ or not. Propensity score matching was used to screen patients according to age, sex, estimated glomerular filtration rate (eGFR) and 24-h proteinuria.

### Treatment

Management of AAV in induction remission and maintenance remission was undertaken mainly following KDIGO guidelines and EULAR/ERA-EDTA recommendations [3, 20]. Patients received glucocorticoids together with cyclophosphamide, rituximab or mycophenolate mofetil (MMF) for induction treatment. Some patients were administered methylprednisolone pulses or plasma exchange. The dosage of glucocorticoid was adjusted by the weight of patients. The initial dose of prednisone was 1 mg/kg/day (or the equivalent dose of methylprednisolone), then the dose was reduced after 2 weeks. We calculated the total prednisone-equivalent cumulative dose and oral prednisone-equivalent cumulative dose. Cyclophosphamide was administered intravenously at 500–750 mg/m^2^ per month and the dosage was adjusted according to the count of leukocytes to maintain it above 4 × 10^9^/L. The rituximab dose was 375 mg/m^2^ of body surface area, once a week for four infusions, or 1000 mg twice a month. MMF was given as 2.0 or 1.5 g/day (for body weight <50 kg) to some patients with non-organ-threatening or non-life-threatening MPO-AAV.

The dose of methylprednisolone pulses was 5–10 mg/kg/day for 3 consecutive days [21]. Alongside this, the dose of HCQ is adjusted by eGFR. If eGFR was >30 mL/min/1.73 m^2^, patients would take 0.2 g/day HCQ orally. Otherwise, the dosage would be 0.1 g/day. Patients of the HCQ group used this drug for >1 month. During maintenance remission, the patients received azathioprine (1.5–2 mg/kg/day) or MMF in combination with low-dose glucocorticoids. The dosage of azathioprine or MMF was adjusted by age, eGFR, side effects and level of white blood cells.

### Definitions and follow-up

Remission included complete remission and response. Complete remission was defined as the ‘absence of disease activity attributable to active disease qualified by the need for ongoing stable maintenance immunosuppressive therapy’. Response was defined as ‘at least 50% reduction of disease activity score and absence of new manifestation’. Treatment resistance was defined as ‘unchanged or increased disease activity in patients with acute AAV after 4 weeks of treatment with standard therapy or a reduction of <50% in the disease activity score after 6 weeks of treatment, or chronic persistent disease defined as the presence of at least one major item or three minor items on the disease activity score list after >12 weeks of treatment’ [4, 22, 23]. Relapse was defined as the recurrence of active AAV after a period of remission [4, 22]. We defined system involvement by biopsy or using previously described criteria [24]. This definition was also consistent with Li *et al*. [25]. For instance, renal involvement was defined by the presence of haematuria (dysmorphic erythrocytes or red blood cell casts, with or without proteinuria) or an elevated serum creatinine level attributable to the disease, or biopsy-proven pauci-immune necrotizing crescentic glomerulonephritis. Ear, nose and throat involvement was defined by clinical evaluation or radiography revealed sinusitis, otitis media, nasal crusting and/or subglottic disease, and other conditions, such as septal perforations. Lung involvement was defined by the presence of pulmonary haemorrhage, respiratory failure, or radiography-proven infiltrates, nodules, or cavities without evidence of infection. End-stage renal disease (ESRD) was defined as a requirement for haemodialysis or peritoneal dialysis for over 3 months or kidney transplantation [26]. eGFR was estimated by the Chronic Kidney Disease Epidemiology Collaboration creatinine equation [27]. We define the time from treatment to the development of ESRD as renal survival and the time from treatment to death as patient survival. Disease duration was defined as the time from symptoms to diagnosis.

Follow-up duration was defined as the time of initial treatment to the occurrence of death or the deadline of follow-up. The date of the last follow-up was 30 December 2023.

### Statistical analysis

SPSS Statistics v.27 (IBM Corp., Armonk, NY, USA) and GraphPad Prism v.9 (GraphPad Software, La Jolla, CA, USA) were utilized to analyse data. We used Shapiro–Wilk test to test normally distributed variables. Continuous data were depicted as mean (± standard deviation) or median (interquartile range). Categorical variables were presented as frequency and percentage. We used 1:1 propensity score matching without replacement to match the experimental group and control group, and the caliper value was set to 0.05. We used pared samples Student's *t*-test or Mann–Whitney–Wilcoxon test to compare continuous data. For comparing the categorical variables, the pared samples χ^2^ test was applied. Kaplan–Meier curves were used to depict survival distribution and log-rank tests were used to verify unadjusted survival differences. A two-sided *P*-value <.05 was considered statistically significant.

## RESULTS

### Demographics and subject characteristics

**Figure 1:**
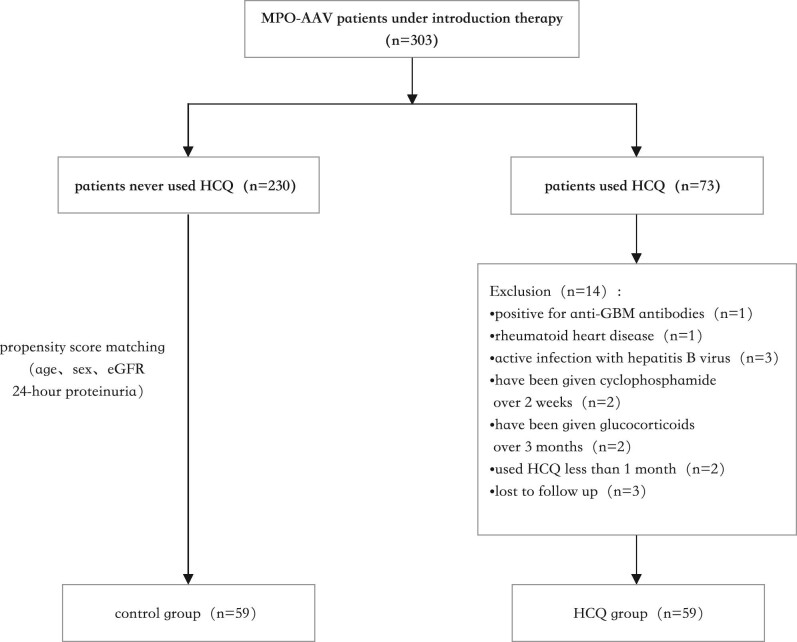
Flowchart of included patients.

A total of 303 patients tested positive for MPO-ANCA between January 2021 to September 2023 in Xiangya Hospital, Central South University (Fig. 1). Seventy-three out of 303 patients used HCQ. After exclusion, 59 patients were included in HCQ group. Patients in the control group were screened by propensity score matching according to age, sex, eGFR and 24-h proteinuria. There remained 59 patients in the control group in the end. As shown in Table 1, no significant difference in age, sex, eGFR, 24-h proteinuria, serum creatinine, BVAS, the titre of MPO-ANCA, and disease duration was found between the two groups. The baseline 24-h proteinuria of HCQ group was 2.03 ± 1.56 g and eGFR was 18.51 ± 14.91 mL/min/1.73 m^2^.

**Table 1:** Baseline clinical characteristics and strategy for therapy.

	HCQ group (*n* = 59)	Control group (*n* = 59)	*P-*value
Age, years (mean, SD)	61.00 ± 12.37	64.78 ± 10.57	.062
Female, *n* (%)	32 (54.2)	30 (50.8)	.851
Proteinuria, g/24 h (mean, SD)	2.03 ± 1.56	1.90 ± 2.64	.076
Initial eGFR, mL/min/1.73 m^2^ (mean, SD)	18.51 ± 14.91	17.17 ± 14.08	.964
Initial serum creatinine, μmol/L (mean, SD)	375.89 ± 215.76	471.24 ± 326.09	.365
BVAS (mean, SD)	14.92 ± 3.43	16.10 ± 4.46	.200
Titer of MPO-ANCA, U/mL (mean, SD)	77.87 ± 39.88	66.91 ± 37.43	.084
Disease duration, day (median, IQR)	121 (45, 287)	91 (50, 293)	.976
Total prednisone-equivalent cumulative dose, mg (median, IQR)	710 (518, 1249)	819 (451, 1272)	.714
Oral prednisone-equivalent cumulative dose, mg (median, IQR)	329 (237, 465)	283 (228, 414)	.115
No. of systems involved (range)	2 (2, 3)	3 (2, 3)	.306
Renal involvement, *n* (%)	56 (94.9)	58 (98.3)	.625
ENT involvement, *n* (%)	2 (3.4)	3 (5.1)	1
Lung involvement, *n* (%)	36 (61.0)	37 (62.7)	1
Treatment, *n* (%)			
MP	10 (16.9)	9 (18.4)	.454
CYC	45 (76.3)	40 (67.8)	.424
PE	8 (13.6)	17 (28.8)	.064
RTX	7 (11.9)	5 (8.5)	.754
MMF	8 (13.6)	16 (27.1)	.115

MP, methylprednisolone pulses; CYC, cyclophosphamide; PE, plasma exchange; RTX, rituximab; SD, standard deviation; IQR, interquartile range.

### Treatment

Glucocorticoids were prescribed for the treatment of all patients in this cohort. As shown in Table 1, there was no statistical difference between the two groups when it comes to total prednisone-equivalent cumulative dose and oral prednisone-equivalent cumulative dose. A few patients were administered both cyclophosphamide and rituximab at different times according to their condition. There was no statistical difference in the percentage of patients who were given plasma exchange and methylprednisolone pulses between the two groups.

### Outcomes and follow-up

**Figure 2:**
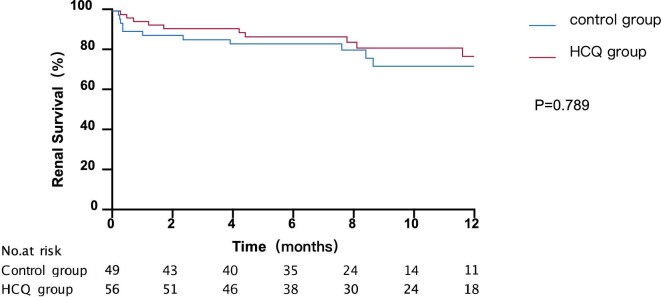
Renal survival Kaplan–Meier curves for HCQ group and control group. Renal survival time (months) refers to the time since treatment. *P* = .789.

**Figure 3:**
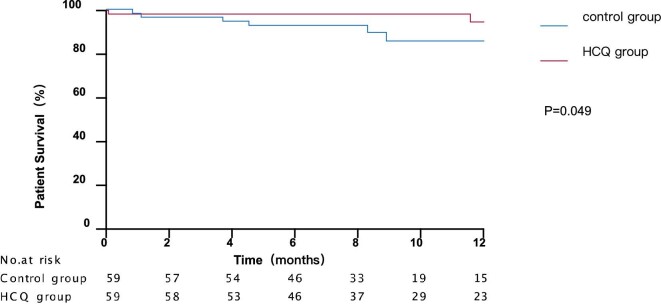
Patient survival Kaplan–Meier curves for HCQ group and control group. Patient survival time (months) refers to the time since treatment. *P* = .049.

As depicted in Table 2, the composition ratio of complete remission, response and treatment resistance in 3 months (*P *= .001) and 6 months (*P *= .021) between HCQ group and control group were statistically different. The rate of total remission tended to be higher in the HCQ group after 6 months, though the difference have no statistic meaning. There was no statistically significant difference in the rate of requirement for renal replacement therapy (25.4% vs 35.6%, *P* = .307), relapse rate (21.4% vs 25.0%, *P *= .709) and death rate (3.4% vs 13.6%, *P *= .109) between the two groups (Table 3). There was no significant difference between the two groups in 1-year renal survival (*P *= .789) (Fig. 2). As depicted in Fig. 3, the HCQ group had better 1-year patient survival compared with the control group (*P *= .049).

**Table 2:** Disease response at 3 months and 6 months.

	3 months			6 months		
	HCQ group	Control group	*P*-value	HCQ group	Control group	*P-*value
Complete remission, *n* (%)	13 (22.0)	1 (1.7)	.001	25 (42.4)	11 (18.6)	.021
Response, *n* (%)	8 (13.6)	6 (10.2)		17 (28.8)	25 (42.4)	
Treatment resistance, *n* (%)	38 (64.4)	52 (88.1)		17 (28.8)	23 (39.0)	
No. of systems involved (range)	(0, 3)	(0, 3)	.024	(0, 2)	(0, 2)	.011
Renal involvement, *n* (%)	46 (78)	58 (98.3)	.002	34 (57.6)	48 (81.4)	.004
ENT involvement, *n* (%)	0	0		0	0	
Lung involvement, *n* (%)	5 (8.5)	7 (11.9)	.774	5 (8.5)	8 (13.6)	.549

ENT, ear, nose and throat.

**Table 3:** Outcomes.

	HCQ group (*n* = 59)	Control group (*n* = 59)	*P*-value
Requirement for RRT, *n* (%)	15 (25.4)	21 (35.6)	.307
Relapse, *n*/*N* (%)	9/42 (21.4)	9/36 (25.0)	.709
Death, *n* (%)	2 (3.4)	8 (13.6)	.109

RRT, renal replacement therapy.

### Safety and side effects

Adverse effects after using HCQ were uncommon during follow-up. One patient felt agitated after taking HCQ for 3 months and the symptom was relieved after stopping using HCQ for 3 days. Two patients developed eye swelling, and two patients had vision loss after taking HCQ for 2 months. These four patients recovered after stopping using HCQ for 1 week. One patient experienced an increase in transaminase which became normal after stopping using HCQ for 1 month. One patient had gastrointestinal reactions and recovered immediately after stopping using HCQ. The side effects of HCQ mentioned above were mild and patients recovered within a certain period after stopping using it.

## DISCUSSION

With the improvement of the treatment regimen, the prognosis of AAV has made great progress. However, it was reported that there remained 10%–25% of patients who were resistant to conventional induction treatment [5–7]. Consistent with our previous study [8], the remission rate of the patients who received the standard induction treatment from our centre was relatively lower. Given that the ANCA type might affect the remission rate, the patients included in this study were all positive for MPO-ANCA, and most patients were positive for PR3-ANCA in Europe or America. Previous studies have reported that the failure to achieve complete remission was related to the antigenic properties of MPO-ANCA itself [28], and MPO-AAV patients had more severe renal lesions than PR3-AAV patients [1]. These might contribute to the lower remission rate of MPO-AAV when compared with PR3-AAV. Importantly, we found that adjunctive use of HCQ initiated concomitantly with induction therapy could increase the remission rate and behave as a positive independent factor for remission in patients with MPO-AAV in this study. Furthermore, patient survival was better in HCQ group than control group.

As a kind of antimalarial drug initially, HCQ has already been used in certain autoimmune diseases successfully. A US prospective multiracial cohort showed that the percentage of 5-year renal damage in patients who used HCQ and did not use HCQ were 20% and 47%, respectively, and the percentage of 10-year renal damage were 38% and 70%, respectively [29]. Meanwhile, it was reported that patients diagnosed with SLE who took HCQ regularly had a lower lupus damage index, less cumulative glucocorticoid usage, lower disease activity and a lower relapse rate [30]. Furthermore, it was shown that HCQ can improve the complete remission rate of patients with lupus nephritis [31]. HCQ has been recommended by the 2021 KDIGO guideline as the basic treatment for patients with lupus nephritis [3]. What is more, one recent study reported that HCQ combined with renin–angiotensin–aldosterone system inhibitors can reduce the proteinuria of patients with IgA nephropathy without major adverse events [32]. HCQ has been added to the treatment of IgA nephropathy in the 2021 KDIGO guideline based on this study [3]. Few reports reported the influence of HCQ on patient survival, the promising result of better patient survival in the HCQ group needs a further randomized controlled trial with a larger sample to verify. The ongoing Hydroxychloroquine in ANCA Vasculitis Evaluation (HAVEN) study (NCT04316494) will provide evidence whether HCQ would improve the patient survival of AAV patients.

The exact mechanisms of action of HCQ remain largely unknown. The plausible mechanisms might be as follows. Firstly, HCQ has the property of being fat-soluble and can easily pass through the cell membrane, and then enter intracellular acidic vesicles like lysosomes. It can change the function of lysosomal vesicles by increasing pH value, thereby reducing the binding of antigenic peptides to major histocompatibility complex II (MHC II). Antigenic peptides–MHC complex is essential for activating CD4^+^ T cells, thus HCQ might reduce immune reactivity against MPO [33]. Secondly, HCQ can reduce serum B-cell activating factor (BAFF) concentration [34]. BAFF was reported to be one of the key factors for promoting the long-term survival of autoreactive memory B cells, and BAFF concentration was positively correlated with the disease activity of AAV [35]. In addition, HCQ can reduce the production of tumour necrosis factor-α, interleukin (IL)-17, IL-6, IL-1β and IL-18, and other cytokines by affecting macrophages and monocytes [36]. These cytokines were reported to be influential in the pathogenesis and relapse of MPO-AAV [37]. Therefore, we assumed that HCQ can increase the remission rate of MPO-AAV by reducing these cytokines. Thirdly, HCQ can block Toll-like receptor (TLR) signalling. It was shown that the alkalinization of lysosomes by HCQ can interfere with the signaling of TLR to antigen-presenting cells and inhibit the activation of TLR, thereby reducing the inflammatory response [38]. Fourthly, HCQ can inhibit the expression of high mobility group box 1 (HMGB1) inflammation signal [39], reduce serum metalloproteinases and increase the levels of their inhibitors [40, 41], which might play an important role in AAV. The mechanism mentioned above may be the potential effects of HCQ in MPO-AAV.

There were several limitations in our study. Firstly, MPO-AAV patients showed good tolerance and compliance with HCQ therapy in our study. However, the sample in this study was still too small to allow for a definitive assessment of the harms of the treatment. Secondly, as a retrospective study, we were unable to monitor patients’ blood HCQ concentrations. In addition, the nature of retrospective study may impact the reliability of HCQ's disease response. Thirdly, the cases in this study were derived from a single centre, so there was selection bias. Therefore, randomized controlled trials with a larger sample size were needed to verify the efficacy and safety of HCQ in AAV.

In conclusion, our data indicate that HCQ combined with standard induction therapy can effectively improve the remission rate of patients with MPO-AAV and it had a relatively good safety profile. Further studies are needed to confirm these effects of HCQ and to investigate its long-term efficacy and safety.

## Data Availability

The original contributions presented in the study are included in the article. Further inquiries can be directed to the corresponding authors.
